# Supplementary figures and images for: Ventral hippocampus to nucleus accumbens shell circuit regulates approach decisions during motivational conflict
Source: PLoS Biol. 2025 Jan 24;23(1):e3002722. doi: 10.1371/journal.pbio.3002722 (PMC11761569; doi:10.1371/journal.pbio.3002722)

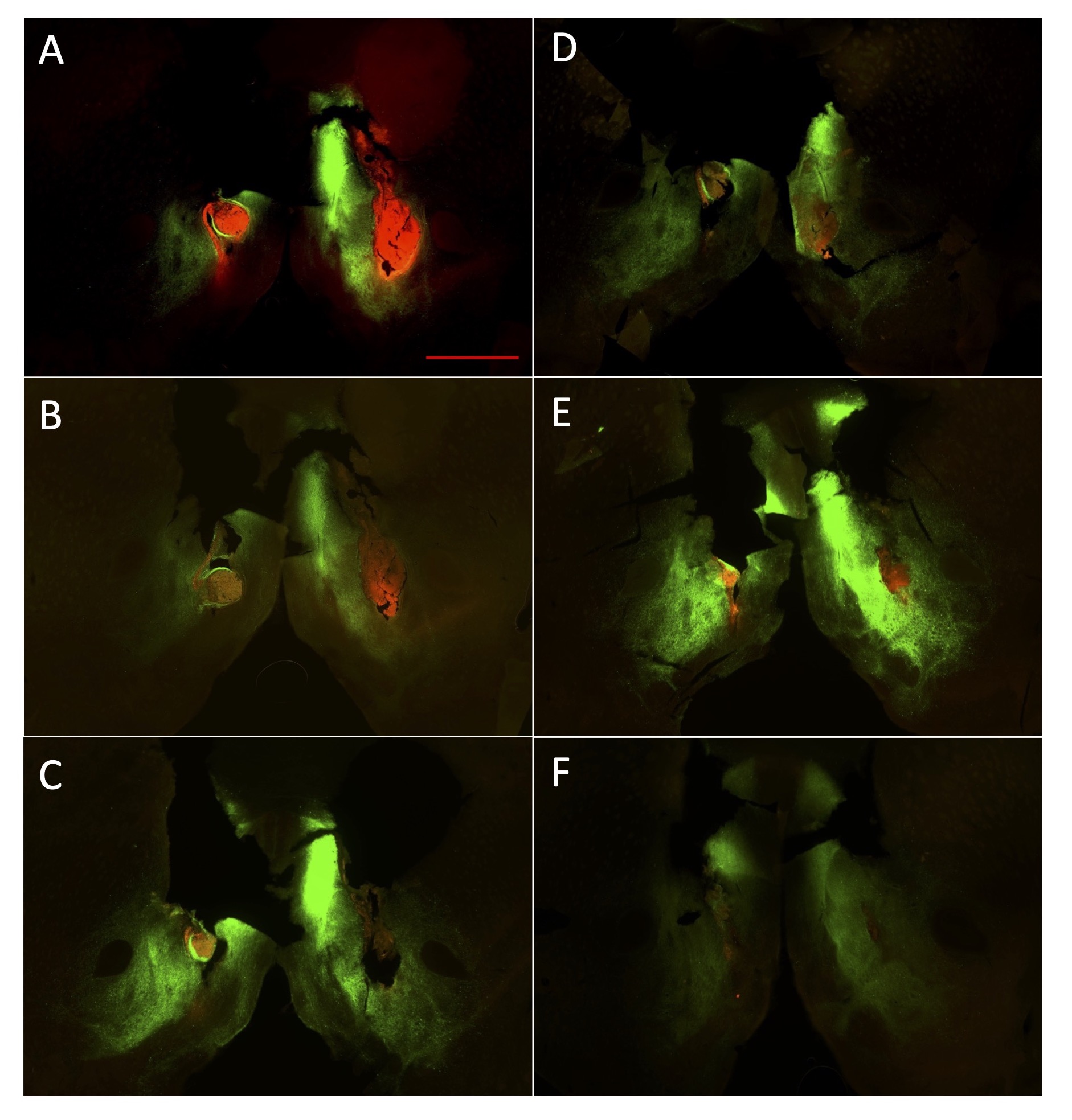

Supplement: S1 Fig — Images (A–F) represent coronal sections along the anteroposterior (AP) axis (approx. +2.1 to +1.0), showing GFP-expressing fibers from the ventral hippocampus (vHPC) terminating in the NAc shell. Bilateral rhodamine-laced CNO infusions (0.3μ l) were localized to the NAc shell and their spread was found to be co-localized within GFP-expressing areas. No encroachment of CNO-rhodamine into overlying areas (e.g., septum) was observed. Scale bar (applicable to all images) represents 1,000 μm. (JPG) [file pbio.3002722.s001.jpg]

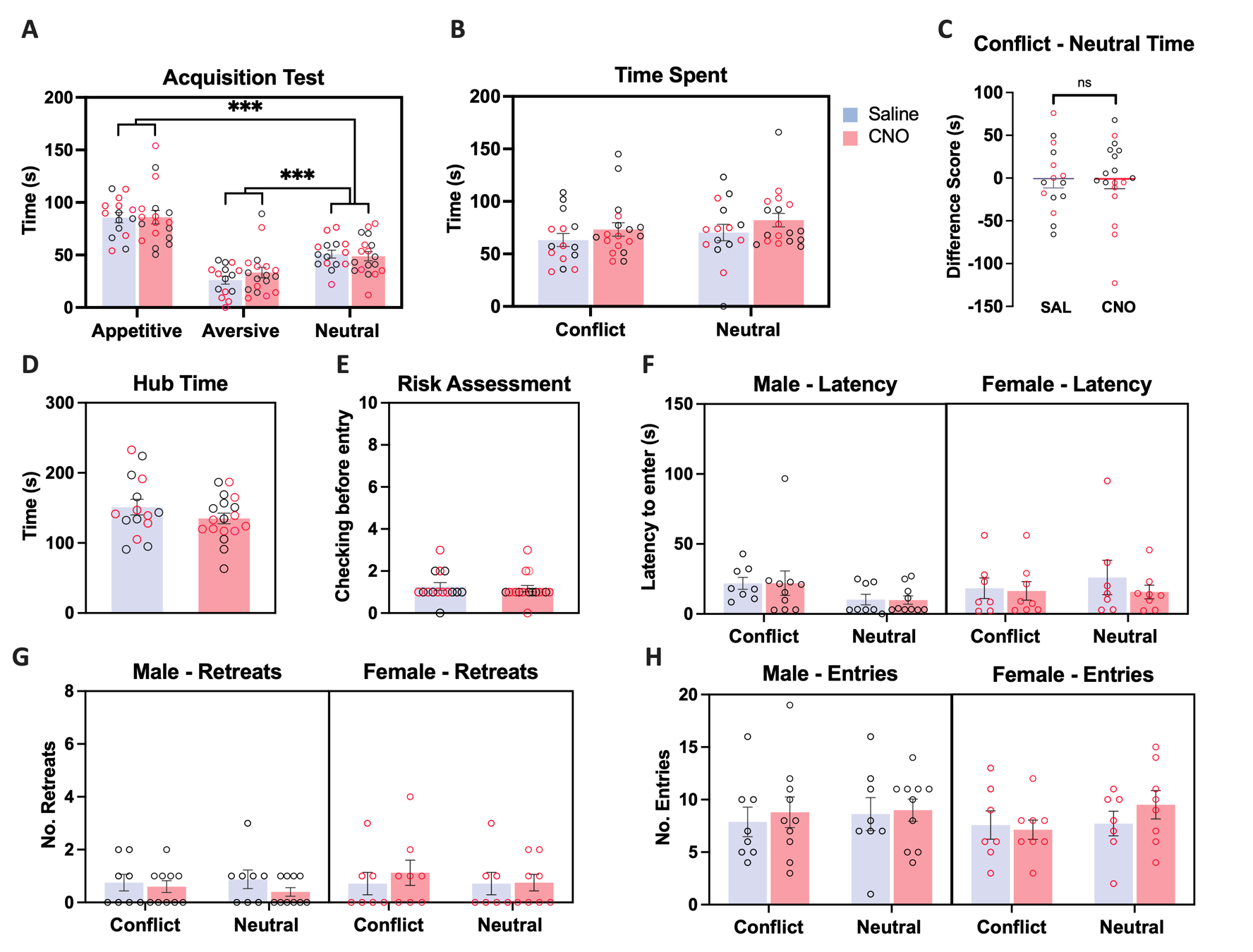

Supplement: S2 Fig — (A) After 2 to 3 rounds of 4 conditioning sessions, 33 animals (GFP Sal: n = 15 (7 females), GFP CNO: n = 18 (8 females), symbols with red borders represent female data) successfully acquired cue-outcome associations, demonstrating a greater amount of time in the appetitive vs. the neutral arm (p < 0.001) and less time in the aversive vs. the neutral arm (p < 0.001). (B) In the conflict test, the time spent in the conflict or neutral cued arms was not significantly different between the CNO or SAL-infused groups, nor between males and females. (C) Neither group exhibited a preference for the conflict or neutral-cued arm. (D) Amount of time spent in the hub (central compartment); (E) Risk assessment (checking) behavior; (F) Latency to enter the conflict or neutral cue arms for the first time; (G) Number of retreats; (H) Number of arm entries were not significantly different between the CNO or SAL-infused groups, or between males and females. Data show means ± SEM. The data underlying this figure are available at: https://osf.io/pwcer/. (TIFF) [file pbio.3002722.s002.tiff]

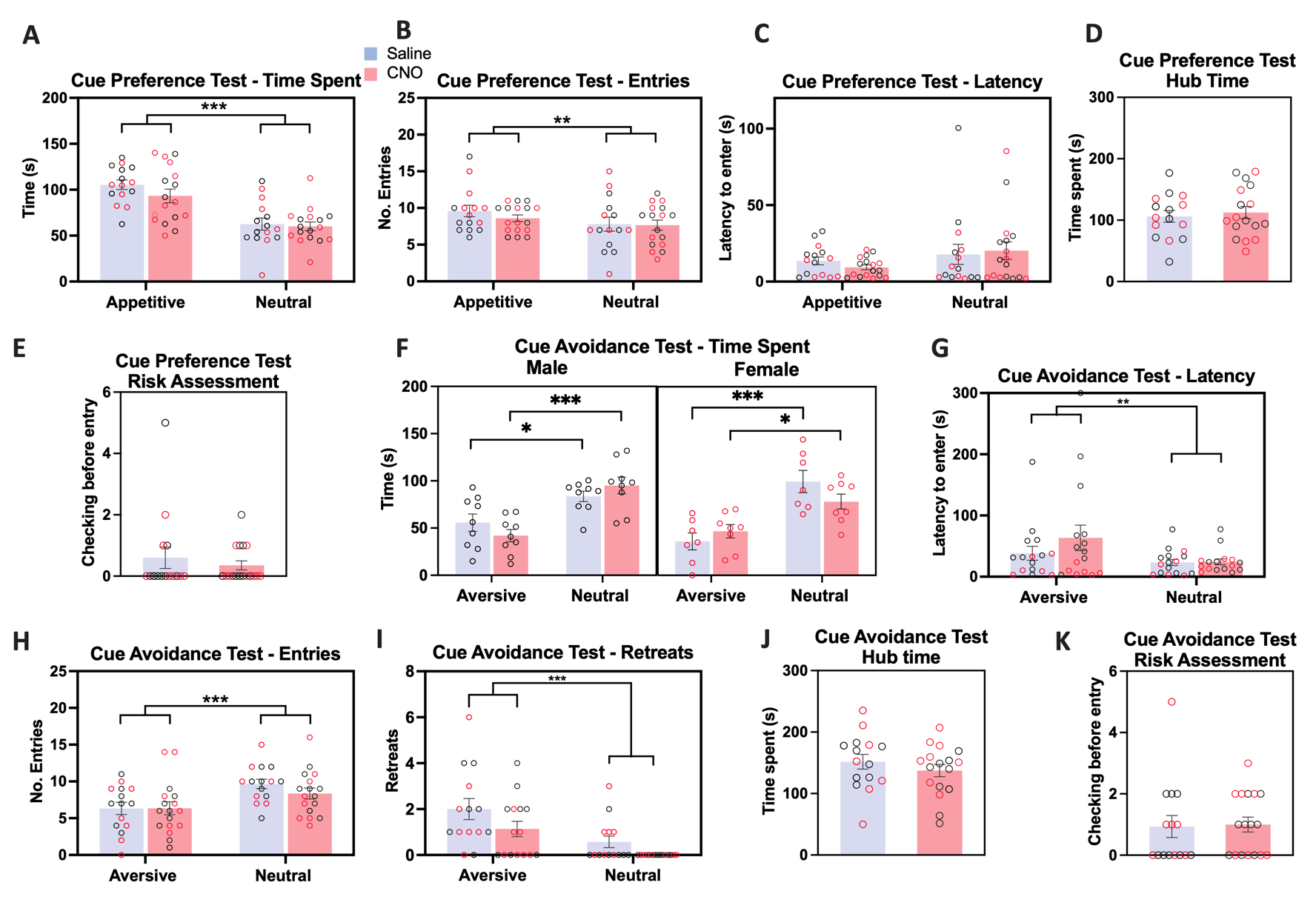

Supplement: S3 Fig — (A, B) GFP-expressing animals (GFP Sal: n = 15 (7 females), GFP CNO: n = 18 (8 females), symbols with red borders represent female data) spent significantly longer time in, and made more entries into, the appetitive vs. neutral arm in the cue preference test, irrespective of drug treatment. (C) No sex or drug treatment difference in latency to enter the appetitive vs. neutral arms was observed. (D, E) There was no difference in the hub time or risk assessment during the cue preference test between any groups. (F) GFP-expressing animals spent less time in the aversive vs. neutral arm in the cue avoidance test. (G, H) Latency to enter the aversive cued arm for the first time was significantly higher compared to latency to enter the neutral arm in both groups, irrespective of sex. (H) Less entries into the aversive arm over the neutral arm in the cue avoidance test were observed, irrespective of drug treatment or sex. (I) The number of retreats observed in the aversively cued arm was significantly higher than in the neutral cued arm. (J, K) There was no difference in hub time and risk assessment behavior between all groups in the cue avoidance test. Data show means ± SEM. * p < 0.01, ** p < 0.01, ***p < 0.001. The data underlying this figure are available at: https://osf.io/pwcer/ (TIFF) [file pbio.3002722.s003.tiff]

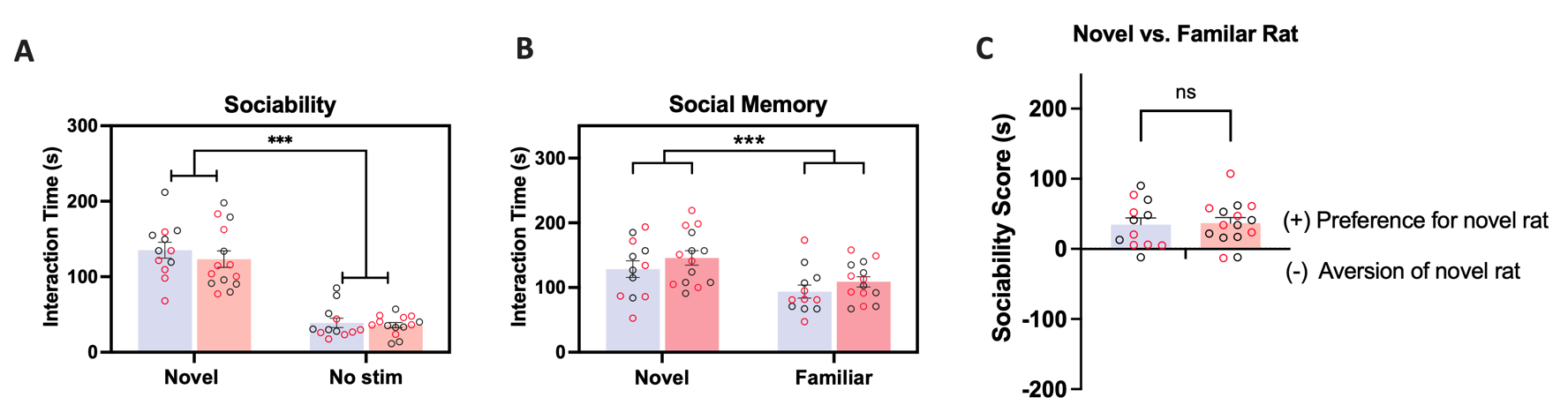

Supplement: S4 Fig — (A) All GFP-expressing animals, irrespective of drug treatment or sex (GFP Sal: n = 12 (6 females), GFP CNO: n = 14 (7 females), symbols with red borders represent female data), spent significantly more time interacting with the novel conspecific over an empty cage, showing preference for social interaction. (B) All animals, irrespective of drug treatment or sex, spent more time interacting with the novel rat over the familiar rat, (C) exhibiting a preference for the novel rat. Data show means ± SEM. ***p < 0.001. The data underlying this figure are available at: https://osf.io/pwcer/. (TIFF) [file pbio.3002722.s004.tiff]

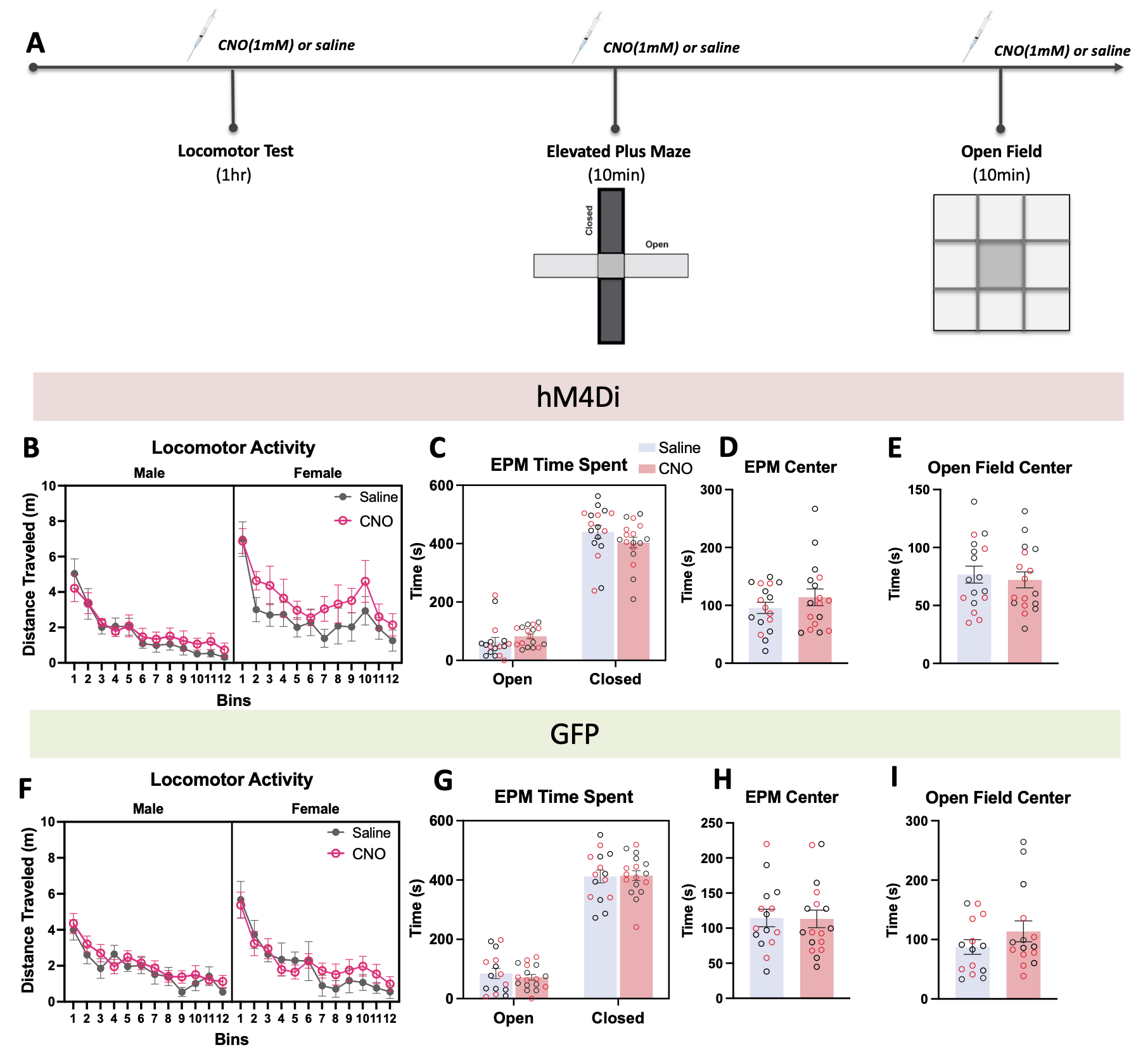

Supplement: S5 Fig — (A) Timeline of final set of experimental procedures involving locomotor activity test, elevated plus maze, and finally, open field test. Animals (hM4Di Sal: n = 17 (7 females), hM4Di CNO: n = 17 (8 females), symbols with red border depict female data) were microinfused with CNO or Saline prior to the locomotor test, and open field test. (B) Females showed elevated locomotor activity overall compared to males, but this was irrespective of vCA1-NAc shell inhibition. (C) All animals spent significantly more time exploring the closed vs. open arms of the elevated plus maze with no group (sex or drug treatment) differences. (D, E) All groups spent equal times exploring the centers of the EPM and open field. (F) The microinfusion of CNO in GFP-expressing animals males and females did not affect locomotor activity (GFP Sal: n = 15 (7 females), GFP CNO: n = 17 (8 females)). (G) All animals spent significantly more time exploring the closed vs. open arms of the elevated plus maze with no group differences. (H, I) CNO microinfusions did not alter the time spent in the center of the EPM or Open field apparatus. Data show means ± SEM. The data underlying this figure are available at: https://osf.io/pwcer/. (TIFF) [file pbio.3002722.s005.tiff]
