# Supplementary material for: Ventral hippocampus to nucleus accumbens shell circuit regulates approach decisions during motivational conflict
Source: PLoS Biol. 2025 Jan 24;23(1):e3002722. doi: 10.1371/journal.pbio.3002722 (PMC11761569; doi:10.1371/journal.pbio.3002722)
Supplement: Attachment — Submitted filename: Response to Reviewers.pdf [file pbio.3002722.s006.pdf]

Patterson et al.,

Response to Reviewers

Reviewer Comments:

Reviewer 1:

In this interesting study, the authors probe a role for vCA1-NAc shell projections in approach-avoidance conflict and find that chemogenetic inhibition of vCA1-NAc shell projections reduces approach of a conflict situation (combined appetitive and aversive cues), increases "risk assessment" behavior, and retreats out of the conflict zone in both male and female mice, and increases the latency to make a decision in male mice. Importantly, these effects were specific to the conflict situation as vCA1-NAc shell projection inhibition did not affect behavior in a conditioned preference task, novelty processing, locomotion, or avoidance. This is a well-controlled study in males and females that reveals an interesting new role of the vCA1-NAc shell projection in conflict avoidance, similar to previous findings on the NAc core.

We thank the reviewer for their positive appraisal of our study and hope that the additional data we have provided have now addressed the reviewers' concerns.

1. It is unclear why hM4Di virus was injected in the vCA1 and CNO infused in the NAc, instead of using a retrograde virus approach to specifically label the vCA1-NAc shell projection. It will be important to confirm that the local CNO infusions in the NAc target specifically the NAc shell and vCA1 terminals therein (e.g., using dye infusions of the same volume used for CNO infusions).

We thank the reviewer for their comment. We opted for the anterograde viral approach combined with direct intra-NAc infusions of CNO, as we believe this to be the most precise method for targeting the vCA1-NAc circuit. Although the dual vector approach is also employed in our laboratory, vCA1 projection neurons are highly collateralized, making transduction to other regions (such as the mPFC, septum, and amygdala) likely when using a retrograde strategy. By delivering CNO directly to the NAc via cannula, we achieve more selective targeting of the vCA1-NAc pathway, minimizing off-target effects.

We have addressed the reviewer's second point regarding the confirmation of the spread to be within the vCA1 terminals within the NAc by taking a subset of GFP-expressing rats and microinfusing a 0.3ul cocktail of CNO and rhodamine, a fluorescent dye. We overcame some technical challenges to generate some images that have now been included in

Supplemental Information (Figure S1), which show the spread of CNO/dye to be discrete, and within the boundaries of vCA1 projections. Please note that we chose to use an overlay of GFP and rhodamine for this procedure, as mCherry signal is typically significantly weaker (than GFP) and we feared that microinfusion of a powerful green dye (such as fluorescein) may obscure the visualisation of terminal mCherry expression.

We would also like to draw the reviewer's attention to the fact that intra-NAc shell (or core) drug infusion technique has been used in our laboratory for over 10 years (e.g., Ito & Hayen 2010, PMID: 21508225, Hamel et al., 2017, 2022 PMID: 28275709, 35351827), and we previously conducted a systematic functional mapping of the action of the same volume of a pharmacological agent (muscimol) dissolved in the same vehicle (saline) by measuring cfos activation in the surrounding radius of the injector tip (Hamel et al., 2017, PMID: 28275709). In that study, we estimated drug spread to be 0.1 mm (radius) per 0.1ul of drug volume injected (0.3mm radius total) with the use of 0.3ul of muscimol. Subsequently, we used 0.3ul of fluorophore-conjugated muscimol to confirm these intracerebral infusion parameters in other brain regions (Schumacher et al., 2018, PMID: 29606418, Riaz et al., 2019, PMID: 28449268), and hence have high confidence that our technique offers highly specific targeting of drug that is largely contained within the shell. The presence of hM4Di/control expression was always confirmed to be within 0.3 mm radius of the injector tip for data inclusion.

2. In Figure 1, the representative image in panel B shows that the vDG and vCA3 are also targeted by the hM4Di injection. Can you exclude that vDG/CA3 do not contribute to the observed behavior effects?

The reviewer is correct in that viral injections in the vCA1 inevitably spread to other subregions of the ventral hippocampus. However, we are confident that the vDG and CA3 regions are not contributing to the observed effects in our study for the following reasons: First, the vDG is not known to have any extrinsic connectivity (outside of the medial temporal lobe) and the lateral septum is the sole recipient of vCA3 outputs (Yeates et al., 2022, PMID: 35688838). Secondly, as explained above, intra-shell injections of CNO via an indwelling cannula allows very accurate targeting of the CNO reaching hM4Di-expressing terminals, and thus the chance of CNO reaching off target sites such as the overlying lateral septum is minimal. We have addressed this concern (and Reviewer 3's) in a paragraph in the discussion (p16).

3. How do the authors reconcile their new findings that specifically vCA1-NAc shell projections regulate conflict approach-avoidance behavior with their previous findings that NAc core is important for this behavior? The cFos data show a reduction in the NAc shell,

verifying neuronal inhibition by hM4Di activation in this region. Given the proximity to the NAc core and its role in avoidance bias in response to motivationally conflicting cues it would be important to assess if activity in the NAc core is also affected by vCA1-NAc shell inhibition.

The reviewer has a valid point here regarding the similarity in the previously reported effects of the NAc core on approach avoidance conflict (note they were GABAR agonists) and the effects reported here. However, we would like to highlight notable differences in the behavioral alterations observed in the present vs. previous study. Firstly, the GABAR mediated inactivation of the NAc core did not significantly affect indices of decision making such as hub time, and hesitancy to enter the arms. Secondly, NAc core inactivated animals also showed clear avoidance of the conflict cue over the neutral cue, with more classical/explicit demonstrations of avoidance, whereas this was not observed with the present pathway manipulation. Thus, the nature of the impairment in manipulating the core vs. vCA1-NAc shell were in fact qualitatively and quantitively quite different.

Following the reviewer's recommendation, we have now assessed cfos density in the NAc core, and have included the data in Figure 1D. Overall density of cfos expression in the core was lower than in the shell in the control saline group, but importantly, we did not see a decrement in density of cfos positive cells in the core, unlike the shell.

4. In Figure 5, female locomotor activity seems to be increased by inhibition of vCA1-NAc projections. What is the main effect of CNO for the RM ANOVA comparing saline vs CNO in the hM4Di vs GFP condition across the 12 time bins in females? It is also not clear from the Figure how many animals were used for this experiment.

Thank you for raising these points. In response to Reviewer 2's request to simplify the graphical representation of our data, we have opted to split the data analysis into hM4Di and GFP groups. Before doing so, we conducted a repeated-measures ANOVA on the female hM4Di group, in line with the reviewer's suggestion, and this yielded a significant main effect of 'Bin' only ($F(5.77,138.52)=25.77$, $p < .001$, $\eta^2 = .97$) with no other significant effects or interactions (e.g., Bin x Drug x Virus ; $F(11,264)=.55$, $p=0.94$, Virus x Drug $F(1,24) = 1.19$, $p=.29$, Drug $F(1,24)=3.54$, $p=0.07$). Visual inspection of the new Figure 5 (hM4Di data only) does appear to show that CNO infusions elicit increased activity in the hM4Di female group. However, we have not been able to confirm this effect with our statistical analysis, with no effect of Drug ($F1,13) =1.60$, $p =.228$) or Drug x Bin interaction ($F(11,143)=.63$, $p = .80$).

We apologise for the oversight of omitting the numbers of animals. All animal numbers for all procedures have now been clearly articulated in a new Methods section entitled 'Overview of Behavioral Procedures' and Figure Legends. The numbers of animals in this experiment were: (hM4Di Sal: n=17 (7 females), hM4Di CNO: n=17 (8 females), GFP Sal: n=15 (7 females), GFP CNO: n=17 (8 females)).

Reviewer 2: This paper examines the role of the ventral hippocampal CA1 projection to nucleus accumbens shell in male and female rats using pathway-specific chemogenetic inhibition to examine the effect of silencing this pathway in tests of learned motivational conflict, cued approach and avoidance, social behavior and innate anxiety-like behavior. Inhibiting vCA1-NAc results in avoidance of an appetitive-aversive conflict cue and increased threat assessment behavior suggesting that behavior is biased towards the aversive association and so that vCA1-NAc is important for biasing towards approach under motivational conflict. This function appears to be specific to conflict in that no effects are observed when testing for approach or avoidance of simple appetitive or aversive cues presented alone. However, it generalizes to social behavior, increasing preference for a familiar vs a novel rats which may indicate that appraising a novel social target involves motivational conflict. The experiments are well designed and well executed and the findings are important. However the manuscript is dense making it challenging to appreciate the work. I have several suggestions for reworking the manuscript to improve the readability and help readers appreciate the significance of this work and a couple of points to address to clarify interpretation.

We thank the reviewer for their positive comments regarding the execution and importance of our study. We hope that the overhaul of the data presentation and analyses has made out paper improve in its readability and that we have addressed all the concerns in full.

- All data are presented in the figures with multiple analyses reported in the text making it difficult to focus on the most important findings. To make this more digestible, the authors could consider moving all the GFP data to supplementary figures. While the authors are to be commended for including controls for both CNO and virus expression (albeit, not the same fluorophore), these data are not essential to interpreting the main findings given that, for most all comparisons, GFP groups show the same result as the DREADD saline control. Additionally, Figure 2 and 3 report multiple behavioral measures for each test although only some show interesting results. Some of these could be moved to supplementary. All of figure 5 could be supplementary material.

Patterson et al.,

We thank you for this comment. We have taken the reviewers' suggestion on board, and have implemented the following changes:

1) All GFP data have been moved to Supplemental Information.
2) To maintain consistency with the graphical representation, where hM4Di and GFP groups are displayed separately, we have simplified the data analysis by conducting it separately for each construct group. We believe that using a 3-way ANOVA, rather than a 4-way ANOVA, provides clearer insights and enhances interpretability.

We have chosen to retain some of the less striking results in Figures 2 and 3, along with Figure 5 as a main figure, as they contribute to providing a more comprehensive picture of the nature of the behavioral deficit/alterations, and provide point by point comparisons across tests (e.g., conflict test, CCP, CCA, Novelty cue preference), which may be valuable for readers. Furthermore, Figure 5 serves to play a key role in demonstrating that the vHPC-NAc pathway is not universally involved in all forms of approach-avoidance conflict.

- P15 line 44-51: 'data not shown' are of interest to the interpretation and should be included as a supplementary figure. This is particularly important given that the result is not significant yet approaching significance (p=.11) and the sample size is modest (n=6).

Thank you for this recommendation. This dataset has now been included in the new Figure 4 for comparisons to be made readily with data in Figures 2 and 3. We have also increased the sample size for this experiment such that we now have n=9 in each group.

- The social behavior tests are not well contextualized until the discussion. Introducing the relevance of these tests (beyond that there is evidence that the CA1-NAc also mediates this function) within the examination of conflict would make the manuscript more coherent.

We agree with this comment, and have included a revised rationale for the social behavioral tests in the Results, and briefly in the Introduction.

- P18 line 32, specify 'social' novelty-seeking given that there is no evidence of novelty-seeking in the cued task

Thank you for this – this has now been edited.

- While in general the behavioral tests are well designed and well controlled, one control

that was not included is a conflict between two conditioned cues of the same valence e.g. two appetitively conditioned cues or 2 aversively conditioned cues wherein the cues predict distinct outcomes with the same valence (e.g. sucrose and chocolate milk or footshock and air-puff). Without this control it is not possible to definitely conclude that it is motivational conflict per se versus conflicting predictions that engage the vHPC-NAC. While I do not think this is necessary to add further experiments to the manuscript, the authors should comment on this limitation within the manuscript.

We appreciate the reviewer's suggestion to include a control involving two conditioned cues of the same valence (but predicting distinct outcomes) as a way to differentiate motivational conflict from prediction conflict. While this would indeed provide additional insight, our study was specifically designed to investigate the engagement of the vHPC-NAc pathway in approach-avoidance conflict, where the interplay between opposing motivational drives (e.g., reward vs. punishment) is critical. Testing two cues with the same valence would primarily address differentiation between outcomes within the same motivational category (e.g., sucrose vs. chocolate milk or footshock vs. air-puff), which is a distinct question.

We would also argue that dissociating conflict behavior driven by motivational factors versus predictive factors is inherently complex. A future approach could involve the use of cues with the same valence to generate approach-approach or avoidance-avoidance conflict. However, such tests may not fully disentangle motivational conflict from prediction-related processes. This would be an interesting avenue for further research, particularly if neural activity could be tracked in real time during decision-making, allowing for dynamic observation of prediction errors and their role in conflict resolution. We have included this acknowledgement in the final paragraph of our discussion.

- Figure 4 E- reformat the graph to compare the groups on the same test rather than comparing across tests.

We have now replotted the original data in Figure 4E and hope that they meet the expectations of the reviewer.

- A couple of other relevant papers to cite
o Role of vHPC-NAc in behavioral suppression:
Yoshida, K., Tsutsui-Kimura, I., Kono, A., Yamanaka, A., Kobayashi, K., Watanabe, M., Mimura, M., & Tanaka, K. F. (2020). Opposing Ventral Striatal Medium Spiny Neuron Activities Shaped by Striatal Parvalbumin-Expressing Interneurons during Goal-Directed

Behaviors. Cell reports, 31(13), 107829. https://doi.org/10.1016/j.celrep.2020.107829

o Role of vHPC-NAc in innate threat behavior and social interaction:
Muir, J., Tse, Y. C., Iyer, E. S., Biris, J., Cvetkovska, V., Lopez, J., & Bagot, R. C. (2020). Ventral Hippocampal Afferents to Nucleus Accumbens Encode Both Latent Vulnerability and Stress-Induced Susceptibility. Biological psychiatry, 88(11), 843-854. https://doi.org/10.1016/j.biopsych.2020.05.021

Thank you for these references – they have now been incorporated into our manuscript.

Reviewer 3: Comments to authors:

In the present study, the authors show that ventral hippocampal (vCA1) projections to the nucleus accumbens shell (NAc shell) regulate approach/avoidance (A/A) conflict. They stated that chemogenetic inhibition of the vCA1-NAc shell projection led the animals to avoid conflict situations with no differences in approach or avoidance components (discrimination/learning). Along with this, the authors showed that inhibition of this projection reduced the approach for social novelty. While these results are straightforward and provide a modest but important characterization of the A/A circuits under decision-making scenarios (e.g. conflict) and built on their previous published findings (e.g. Yeat et al., 2022), the manuscript was difficult to read and follow. Problems with the organization, rationales, figure/experimental design, male/female distribution, subset of groups, statistical design, and interpretations detract from the impact of the study to be considered for publication in Biological Psychiatry.

We thank the reviewer for recognising the importance of our study, and for their careful reading of our manuscript. We have made substantial changes to the manuscript to improve the readability and transparency in reporting of animal numbers/procedures and hope that the reviewers' concerns have been addressed in full.

Major
1. Title: the authors did not highlight the main finding of the study: inhibition of the vCA1-NAc shell projection biases the animals to avoid conflict situations...

We had felt that a descriptive title that explains the function of the vCA1-NAc was more informative than a description of the finding of the study. However, if the reviewer/editors at PLOS Biology prefer one style over the other, we would be happy to change it.

2. Please provide a schematic portraying the timeline of all phases, CNO injection days, and the days that additional behavioral readouts were incorporated (e.g. social, EPM).

The figures are laid out with a timeline at the top of each figure, and the behavioral tests were conducted in the order in which they are presented. The time point of CNO or saline injections is also indicated with a syringe on the timeline. We have also added a paragraph in the Methods to explain the order of testing and animal numbers at different stages of the experiment (including explanations of attrition/data exclusion).

3. Please incorporate the importance (rationales) of the behavioral phase (e.g. Cue preferences/novelty) in the methods. This would help to follow the logic.

Since the Results section comes before the Methods section, we have provided a brief rationale for each study at the beginning of the respective procedures. Additionally, we have made sure to further clarify these rationales within the Methods section for consistency and clarity.

4. Related to point 2- please add the number of animals per phase. Because every phase seems to have a different number of animals, adding the exact numbers in the figure (or figure legend) would help a lot.

The reviewer's point is well taken. We have now updated the figure legends to include the animal numbers for each phase of the study, and the same information is also in the Methods section entitles "Overview of Behavioral Procedures'.

5. Related to point 4- Because every phase seems to have a different number of male/female rats, adding the exact numbers in the figure (or figure legends) and/or explicitly saying it in the Result or Method section would benefit the reader.

As per our response to Point 4, we have now provided these numbers (males, females) in the figure legends and Methods section.

6. In the Social tests (methods) the use of a subset of animals (n=8) was mentioned, however, in the figure legend, other numbers were provided. Please clarify.

To clarify what was written in the Methods, data from 8 animals (early batch of animals) were excluded from the sociability and social memory test since there was a procedural

difference in phase 1 of the social tests (they were exposed to two novel animals (rather than 1) in the sociability test). The final numbers of animals included in the data analysis were as reported in the legends of Figure 4, and Methods section.

7. Were the locomotor and anxiety tests performed on the same animals (previously receiving the A/A tests) or in naïve animals only (no previous A/A experiences)? If the latter is the case, the rationales behind these tests are not completely accurate (page 16-line 49; page 17-line 10; & methods page 8), where these behavioral readouts would solely address the role of the vCA1-NAc shell projection in locomotion and anxiety. On the other hand, it seems that the OFT was performed on conditioned animals (but this didn't happen for EPM?). If the first is true, please discuss the potential problems of administering multiple CNO infusions and multiple behavioral tests on the same animals (e.g., the impact on further behavioral outcomes and cFos signaling). Please clarify/discuss.

The locomotor and anxiety tests were conducted in the same animals that had undergone the AA conflict and social preference and memory tests. The reviewer raises a valid concern that is generally overlooked by researchers in the field. Behaviorally, we did not, and have not, observed any undesirable effects of repeated injections of CNO/DREADDs activation in the current study and previously published DREADDs experiments (e.g., Cavdaroglu et al., 2021, PMID: 33154580, Yeates et al., 2022, PMID: 35688838), as have other studies (e.g., Tran et al., 2020, PMID: 33080350). We routinely conduct positive control experiments at the end of our experiments, and have replicated expected effects (e.g., effect of ventral hippocampus inactivation on elevated plus maze or novelty suppressed feeding) in similarly long studies with repeated injections. As a rule, we also limit the number of intracerebral infusion to a maximum of 10 due to the possibility of incurring local tissue damage, and in the present paper, each animal received a maximum of 6 infusions. Of note is that we validate the neural inhibition of targeted areas/circuits using cfos staining at the end of every experiment, and observe expected reduction in cfos activation after CNO infusions.

8. Stats section and Results- the use of two or three-way ANOVAs for the sex variable is convoluted throughout the manuscript, in part because the figures are not directly comparing/depicting the sex variable. Please make this more clear... maybe add supplementary figures (this would be key) for these statistical analyses. This would help to visualize the results and analyses.

We apologize for any confusion. We have now clarified in the Methods section (Data Analysis) that all analyses included Sex as an independent variable. To ensure consistency

between the statistical results and graphical presentation, we organized the figures so that male and female data are plotted separately only when significant effects or interactions involving Sex were found. When no sex differences were observed, the data for males and females were combined and represented using different colors (black borders for males, red for females). Given the already data-heavy nature of our experiments, we decided to follow Reviewer 2's suggestion to separate the graphs for hM4Di and GFP groups. However, separating the data by sex for each behavioral measure would result in a significantly higher number of figures, which may not reveal any meaningful differences. To avoid unnecessary complexity, we opted for the current presentation.

9. Although the authors reported a significant decrease in cFos activity after CNO exposure, this was not necessarily supported by the present data/images (Fig. 1D). Please also provide the animals used in this analysis and the number of animals. ANOVA results were reported but the figure displays two columns (male/female combined; missing t-test and/or posthocs values?). Please clarify (e.g., explicitly say that no m/f differences were observed, then results were collapsed).

We have now added the additional information regarding the number of animals and clarified in the text that there were no differences in female/male data (please note that we did report the F_{Sex} stats in the first paragraph of the Results section).

10. Fig. 2- it's hard to see/follow the sex comparisons; incorporating supplementary figures would help. For instance, sex differences in latency times are highlighted but no direct comparisons or figures were shown to support this.

We appreciate the suggestion to clarify the sex comparisons. Since there was no overall main effect of Sex, the primary difference in the latency data was that males showed a selective increase in latency to enter the conflict arm following CNO infusion, whereas females did not. We have now addressed this by including visual comparisons in the figure, clearly indicating both significant and non-significant data pairs to support the sex-specific effects.

11. Fig. 2H- Difficult to follow these stats in the figure. Why would the animals have more retreat for the neutral area (this is not an aversive area)? A virus effect?

The reviewer raises a good point that the pathway inhibited animals appear to be generalizing aversion to the neutral cue. As per Reviewer 2's suggestion, we have now separated the graphing and analysis of GFP and hM4Di groups and moved all GFP graphs to

supplement information. However, we would like to report that there was no main effect of virus (p=0.195) in this data set (4 way ANOVA with virus as one of the between subjects factors).

12. Fig. 2i-J (page 14-lines 1-15)- generating a separate figure reflecting this male/female comparison/difference would help. In the current version, it's hard to visualize.

We hope that the reorganization of the figures (separating the hM4Di and GFP data) has now made this sex comparisons easier to identify. In a further attempt to clarify the sex comparisons, we have also added significance and non-significance indicators directly to Figure 2K to show how the male and female data differ.

13. Related to point 12- sentence stipulating: "albeit CNO induced inhibition of the vCA1-NAc circuit did not induce any significant changes in the number of entries into the conflict and neutral arms" So, this seems to be only in saline (suggesting natural sex differences?).

The statistical analysis shows that females enter the neutral arms more readily than the males in both saline and CNO-infused groups, which could indeed speak to sex differences. Preferred entry into the neutral arm could be an index of females being more avoidant of the conflict cues as well. Interestingly, however, this sex difference was only apparent in the hM4Di group, and not the GFP group. Based on this comment, and some others, a brief discussion on the sex differences has been included in the discussion.

14. Page 14 lines-17-59 and page 15 lines-1-29 (Fig 3)- the order of the results did not match with the order of the figures panels (everything was mixed). This was extremely difficult to follow. Mention B before C; panel D was not mentioned. Please provide interpretations (suggestions) for the findings in the results.

We apologise for the confusion with figure panels. We will ensure that these mix-ups are corrected in the revised version, and explanations for the cue preference and avoidance test results will be provided.

15. Page 15 line 14/15 (Fig. 4)- what subset of animals: m/f? showing lower/higher conflict? Please justify/clarify. Talk about Figure 4C. Page 15 line 48/49: "not shown"; Consider a supplementary figure or show the values/SEM.

In the original submission, we had a total of 12 rats in this sub experiment, with 4 males and 8 females. In the revised paper, we have added 6 additional animals to this group (given time and resource constraints) such that we now have a total of 18 rats in this experiment with 8 males and 10 females. We have also included the data ‘not shown’ in Figure 3, as requested by Reviewer 2 as well.

16. Page 16 second paragraph (fig. 4). Was this test performed in male and female rats? Please clarify and discuss this.

Yes, all our experiments were performed with male and female rats, as per our response to 15. Male and female data are now clearly differentiated in the graphs.

17. Page 16 last/third paragraph- page 17 (fig. 5) please provide the full timeline of when these readouts were performed (related to point 2; see above).

The tests shown in Figure 5 were conducted at the end of the experiment – and we have now clarified the timeline as per our response to Point 2.

18. Discussion- page 18 - first paragraph first sentence- I'm wondering whether the animals had a deficit in decision making or they just decided to avoid the conflict (the arm signaling conflict).

Thank you for a request for clarification here. We will rephrase this sentence to: 'In the present study, chemogenetic inhibition of the vCA1-NAc pathway resulted in **a decision bias to avoid** in the face of motivational conflict.' We believe that the profile of behavioral deficit overall points to a decision-making deficit than an avoidance deficit *per se*.

19. Page 18 second paragraph- line 54- "indecision". Was this not a decision to avoid the conflict arm? You can potentially discuss the limitations of this task on capturing these behavioral biases.

A few converging behavioral observations support our statement that animals were exhibiting 'indecision' in the face of a choice between approaching a neutral cue vs. conflict cue, and not purely a decision bias to avoid the conflict cue. First, we observed an increase in hub time in the absence of any preferential exploration of the conflict vs neutral arms, which we interpret as an increase in deliberation time (see our responses to more points below). Notably, we also observed an increase in 'looking' behavior, which is akin to a stretch-attend posture when animals encounter an approach avoidance conflict. Finally, male vCA1-NAc shell inhibited animals showed significantly increased latencies to enter the conflict (but not neutral) arm, which appears to indicate that the decision hesitancy may be directed at the conflict cue.

Minor

1. Abstract (conclusion)- please mention the main finding (conflict).

The main finding was stated earlier in the abstract and hence we had wanted to avoid repetition in the concluding sentence.

'Chemogenetic inhibition of the vCA1-NAc shell circuit resulted in animals exhibiting increased decision-making time and avoidance bias specifically in the face of motivational conflict, as the same behavioral phenotype was absent in separate conditioned cue preference and avoidance tests.'

2. Please provide the viral titers.

This information has been added.

3. Please provide the CNO source (e.g., product number, company).

The requested information has been provided.

4. Please discuss the potential problems of multiple CNO infusions/inhibitions.

Please see our response to Point 7 above.

5. Cell imaging and counting (Page 10-line 25)- were the two images/sections selected based on the CNO expected spread? The radio of inhibition should reflect the CNO spread. Please clarify or add a limitation/caveat.

The reviewer is correct that the two images were meant to show representative areas in the NAc from which we conducted the cFos cell counting. However, they were not intended to reflect CNO spread. Nevertheless, we have now replaced the images with new images that show cfos expression in the core as well as the shell. Additionally, in response to the reviewer's comment, and as requested by Reviewer 1, we have now added additional images of CNO spread (using CNO+rhodamine) in Figure S1 (supplemental info), which indicate that the extent of CNO spread is limited to areas of GFP expression.

6. The viral spread displays strong labeling in the septal/shell area (vCA1 terminals; as previously published by the group); please discuss the potential impact of this projection in the present results (potentially due to the CNO spreading in the adjacent septal areas?)

The reviewer raises a valid point, but based on the positioning of the guide cannula (as seen in Figure S1), we are confident that the observed behavioral effects are specifically due to local CNO injections into the NAc. Furthermore, the effects of inhibiting the vCA1 terminals in the septum are in fact opposite to those observed in the current study (Yeates et al.,

2022, PMID: 35688838). We have now added a paragraph in the Discussion (p16) to address the reviewer's concern.

7. Fig. 2C (GFP-neutral)- female dots are missing?

Thank you for pointing this out. We have added the female dots to the graph in the revision.

8. Page 13-line 16; what does the increased time in the hub area suggest? When no differences were observed in the time spent in the neutral and conflict arms. Please provide an interpretation in the results or discuss it.

We interpret the time spent in the hub, or central compartment to be a decision-making parameter, and therefore increased time spent therein reflects slowed decision making and/or increased deliberation time before entering the arms. This is in line with the central compartment in the elevated plus maze being similarly regarded as an area of choice point, where animals exhibit vicarious trial and error behaviors, which include 'stretch-attend postures' (synonymous with our 'looks' measure) as an index of risk assessment (Redish, 2016, PMID: 26891625). We will include an extended discussion on this literature in the discussion.

9. Page 13-line 34; more "looking" behavior suggest??

As explained in the point above, we consider 'looking' behavior to be synonymous with 'stretch-attend postures' that animals emit as they are making a decision to enter one of the cued arms.

10. Fig. 2G. It seems that you have a CNO effect on females, GFP conflict; please address/discuss it.

Multiple comparison tests (corrected) did not reveal significant CNO effects in the GFP females: conflict: sal vs CNO: p=.44, neutral: sal vs CNO: p=.28

11. Fig. 3A and 4- please add arm labels.

Thank you for pointing this out – we will add arm labels to the schematic diagrams.

12. Page 15 line 56- provide a reference.

We will likely be replacing this sentence with a more appropriate rationale (as per Reviewer 2's suggestion).

13. In the discussion, you could consider adding/suggesting chemogenetic activation (Gq) for future steps.

While we agree that this may be an interesting future direction, we are also cautious about applying this approach in general for the following reasons. The use of chemogenetic inhibition demonstrates that a neural circuit is necessary for a certain behavioral process, as we have demonstrated in the present study. However, chemogenetic activation requires that a certain threshold of activation is reached before the sufficiency of that brain region can be established in a certain behavior, and thus, the effects of inhibiting and activating a certain brain area/pathway need not be expected to have opposite effects on behavior. In fact, there are studies that demonstrate that optogenetic silencing of the ventral dentate gyrus has no effect on anxiety, as measured by the elevated plus maze, whereas optogenetic activation of this area induces decrease in anxiety, rendering interpretations of the divergent effects of inhibition and activation very difficult (Kheirbeck et al., 2013, PMID: 23473324).

14. Page 16 lines 18-21- please provide an interpretation of the importance of these results.

Unfortunately, we are not able to find the passage the reviewer is referring to without further details of the results the reviewer wishes for us to discuss (the original manuscript did not contain line numbers).

15. Page 17-line 47 "inhibition impaired sociability" Does this also apply to animals with the A/A experience (conditioning and conflict experiences)?

All animals experienced the AA conditioning and conflict tests prior to the sociability tests. This has now been made clear in a new paragraph detailing the timeline of procedures in the Methods (p17).

16. Page 18 second paragraph- difficult to see the link with dopamine (D1 pharmacology).

Thank you for this comment. We have now removed this reference.

17. Page 18 second paragraph- line 59- "aversion". Hard to say if this is aversion, you could

also say cautious/exploratory/insecurity behaviors (and mention the risk assessment behaviors)

Thank you for this comment. We can certainly add ‘show of caution’ to our interpretation of ‘retreat’ behaviors. We have leaned towards the aversion interpretation as these retreat behaviors occur during exposure to an aversive cue as well. However, we agree with the reviewer that it may also be considered as part of a repertoire of risk assessment behaviors as they are occasionally emitted in the neutral arm as well.

18. Page 19- line 5- "altogether pointing to a decision-making deficit"; or the decision was to stay in the hub area and not explore the conflict arm?

As with our response to Major point 18 above, we have revised this statement to be more specific: ‘altogether pointing to an avoidance decision bias’.

19. Page 20 - line 4- "avoidance of the novel rat". Or avoid the conflict for social scenarios as well?

Thank you for this point. Yes indeed, this could be conceived as the avoidance of a social conflict situation, which we discuss in the previous sentence.

20. Page 21 - line 33- for caudal NAc including core/shell or only shell? please clarify/discuss.

Thanks for pointing this out – this has now been corrected to ‘caudal NAc core’.

21. A brief discussion comparing males vs females in A/A tasks would be timely and meaningful.

Thank you for this suggestion. As mentioned above, we will include a discussion on sex differences in approach-avoidance conflict.

22. Fig. 5A. What was the time between the locomotor and anxiety tests (time in between; 24hr?)

A minimum of 48hrs were given in between individual tests for the drug to wash out. This information has now been included in the Methods (p17).

23. Fig 2 E & D- when these analyses were made?

We are unclear on the specific aspect of the analyses that the reviewer is referring to here. Retreat behaviors and hub time were scored using video recordings.

24. Figure legend 1B: axonal transport of the viral load instead of axonal transduction?

This has been edited as per the reviewers' suggestion.
