# Supplementary material for: Ventral hippocampus to nucleus accumbens shell circuit regulates approach decisions during motivational conflict
Source: PLoS Biol. 2025 Jan 24;23(1):e3002722. doi: 10.1371/journal.pbio.3002722 (PMC11761569; doi:10.1371/journal.pbio.3002722)
Supplement: Attachment — Submitted filename: Response to Reviewers.docx [file pbio.3002722.s007.docx]

**Response to Editors and Reviewers**

1) TITLE: While we think the current title does a good job capturing the message of the study, I have been wondering if this might be streamlined a bit to make it more accessible to our broad audience.  As an example, if you agree (and feel this is supported), we would suggest you change the title to something like:

   "A neural circuit between the ventral hippocampus and nucleus accumbens regulates approach decisions during motivational conflict"

   I am happy for you to refine further

We have made the following change to the title, and hope this is more accessible and acceptable.

‘Ventral hippocampus to nucleus accumbens shell circuit regulates approach decisions during motivational conflict’

2) FIGURES: Sorry for only raising this now, but please note that PLOS Biology Short Reports can have a maximum of 4 figures, while the current work has 5. I therefore request that you streamline the figures a bit to meet this requirement. I suspect the easiest way to condense the data into 4 figures would be to just combine 2 of the existing figures? Feel free to reach out to us if you would like to discuss this further.

We have moved the original Figure 5 (innate anxiety data) to the supplementary information as the data were not central to the main story.

3) ETHICS STATEMENT: Thank you for providing an ethics statement in your methods section. Please update this to include the approval number for the protocol, approved by the IACUC at University of Toronto.

This number has now been included.

4) DATA: Thank you for depositing the underlying data related to your study to OSF. For the most part this looks good to me but can you please either update the file on OSF, or add a readme that more clearly spells out which figures the data relates to? Please also add a sentence to each figure legend pointing readers to this file. (For example, you can say "the data underlying this figure can be found at  <https://osf.io/pwcer/?view_only=46274147a9214b6a994322b90f67d378>")

The data sheet has been reorganized to delineate a clear link between the Figures and the dataset.

5) CODE: Per journal policy, if you have generated any custom code during the course of this investigation, please make it available without restrictions. Please ensure that the code is sufficiently well documented and reusable, and that your Data Statement in the Editorial Manager submission system accurately describes where your code can be found.

We did not generate any custom code for any part of the investigation.

Please note that we cannot accept sole deposition of code in GitHub, as this could be changed after publication. However, you can archive this version of your publicly available GitHub code to Zenodo. Once you do this, it will generate a DOI number, which you will need to provide in the Data Accessibility Statement (you are welcome to also provide the GitHub access information). See the process for doing this here: <https://docs.github.com/en/repositories/archiving-a-github-repository/referencing-and-citing-content>

Reviewer #3: While I recognize the authors' effort to improve the paper, there are still few observations/limitations and additional comments to be considered and addressed to improve the manuscript in general (for this or other journal). 

1-Fig1S (related to Reviewer 1; Point 1)- the authors should just state that a limitation may include CNO spreading into the nearby regions (i.e. core and septum). it's also clear that vHPC/CA1 projects to the NAc core (based on the terminals (green labeling)).

- In response to the reviewer’s points, we have made the following changes to the opening paragraph of the Results section (page 4):

1) make reference to the fact that vCA1 projects to the boundary between core and shell (called the ‘shore’) in addition to the shell, and

2) although we believe we have provided strong evidence that the spread of the drug is confined to targeted areas with vCA1 terminals, we have added the following statement:
“*However, it is acknowledged that, despite the efforts to confirm localized diffusion presented herein, a limitation of this approach is the possibility of CNO spreading into adjacent or overlying regions due to individual variability; albeit it should be noted that previous work from our laboratory has demonstrated that chemogenetic inhibition of vCA1 terminals in the lateral septum produces effects opposite to those observed here on AAC^17^.*”

2- Figs2-3 and 5- Unclear: would it be better use DREADDS (hM4Di-mCherry) or empty vector (mCherry or GFP) groups both with CNO (as in Fig 1)?

- We did employ a design in which we had both DREADDs and empty vector groups with CNO and Saline (control). Thus, we had animals assigned to 4 groups: DREADDs-CNO, DREADDs-Saline, GFP-CNO, GFP-Saline. However, at the request of Reviewer 2 from the previous round of review, we split up the data presentation such that all GFP (control) data/figures are now in the supplementary information bar a few graphs in which we make direct comparisons between the groups. Our experimental design has now been made explicit in two places in the manuscript – in the Results and Methods section.

3- Related to Reviewer 3; Point 7- As the authors mentioned "Of note is that we validate the neural inhibition of targeted areas/circuits using cfos staining at the end of every experiment, and observe expected reduction in cfos activation after CNO infusions". Would be useful to show this in the supplement?

- We apologise that this was not made clear in our original response, but this cfos validation data is presented in Figure 1D where we successfully demonstrate the effectiveness of the circuit manipulation by verifying that cfos activation (proxy of neural activity) is reduced in the NAc shell in CNO-infused, but not saline-infused hM4Di animals.

4-  Related to Reviewer 3; Point 16 (Fig4)- I can't still find the m/f labels figures/panels.

- The male/female data are differentiated based on the colour of the symbols (red for females). They were not plotted separately as their data were found not to be statistically significant. This is now explained in Page 28.

5- Related to Reviewer 3; Point 23- what phase? (mix valence, acquisition or conflict).

- Thank you for clarifying the original question – we recorded the retreat behaviors and hub time only during the conflict and cue preference/avoidance tests after the animals were under the influence of CNO or saline.
